# Developing neural stem cell-based treatments for neurodegenerative diseases

**DOI:** 10.1186/scrt461

**Published:** 2014-05-30

**Authors:** James A Byrne

**Affiliations:** 1 Department of Molecular and Medical Pharmacology, 650 Charles E. Young Drive South, 23–120 Center for Health Sciences, University of California, Los Angeles, CA 90095, USA; 2 Eli and Edythe Broad Center of Regenerative Medicine and Stem Cell Research, 615 Charles E. Young Drive South, University of California, Los Angeles, CA 90095, USA

## Abstract

Owing to the aging of the population, our society now faces an impending wave of age-related neurodegenerative pathologies, the most significant of which is Alzheimer’s disease. Currently, no effective therapies for Alzheimer’s disease have been developed. However, recent advances in the fields of neural stem cells and human induced pluripotent stem cells now provide us with the first real hope for a cure. The recent discovery by Blurton-Jones and colleagues that neural stem cells can effectively deliver disease-modifying therapeutic proteins throughout the brains of our best rodent models of Alzheimer’s disease, combined with recent advances in human nuclear reprogramming, stem cell research, and highly customized genetic engineering, may represent a potentially revolutionary personalized cellular therapeutic approach capable of effectively curing, ameliorating, and/or slowing the progression of Alzheimer’s disease.

## 

The recent discovery that neural stem cells (NSCs) can effectively deliver disease-modifying therapeutic proteins throughout the murine brain [1] provides hope for millions worldwide who suffer from neurodegenerative disease. In this commentary, I will attempt to place this important research in a broader historical, technological, and scientific context.

Since the beginning of recorded history, researchers have sought therapeutic biologics capable of rejuvenating our bodies and minds [2]. Gilgamesh’s ancient rejuvenation research proposal is as follows:

*There is a plant that looks like a box-thorn,it has prickles like a dogrose, and will prick one who plucks it.But if you can possess this plant,you’ll be again as you were in your youth.*

This plant, Ur-shanabi, is the ‘Plant of Heartbeat’,

 *with it a man can regain his vigour.*

To Uruk-the-sheepfold I will take it,

 *to an ancient I will feed some and put the plant to the test!*[2]

This goal has become a critical issue in modern times as technological advances have permitted remarkable increases in both human life expectancy and the percentage of the adult population surviving into old age [3]. However, the discovery of technologies to extend our lives has far outpaced our ability to maintain our minds as we age, and our rapidly aging population means we now face an impending wave of the age-related neurodegenerative pathology, Alzheimer’s disease (AD). Forecasts indicate that by 2050, in the United States alone, over 13 million people will suffer from AD [4]. Novel cellular therapies built on new technologies are beginning to be developed for a broad range of diseases, and AD offers a potentially promising indication.

NSCs, derived from pluripotent stem cells, are capable of improving cognitive function in preclinical rodent models of AD [5,6]. However, NSCs alone do not remove the amyloid-beta (Aβ) plaques, which (alongside tau-based neurofibrillary tangles) may represent a partial cause of the AD pathology. Neprilysin is a potent proteolytic enzyme that is the rate-limiting enzyme involved in the degradation of Aβ and is found at decreased levels in AD brains. Although virally mediated delivery of neprilysin can reduce Aβ pathology in murine brains, the potential application of this viral approach for much larger human brains is restricted because of its limited radius of infectivity. NSCs offer a potential solution to this clinical bottleneck of narrow viral delivery. NSCs are highly migratory in nature and thus may be suitable for delivering secreted neuro-therapeutics throughout the entire human brain, an activity that NSCs could perform alongside their other putative cognitive functional improvements. These NSCs could be genetically modified to secrete neprilysin or other therapeutic transgenes (or both) for the treatment of AD and other neurodegenerative pathologies, such as Parkinson’s disease, stroke, and amyotrophic lateral sclerosis.

Blurton-Jones and colleagues have now demonstrated that NSCs can effectively deliver disease-modifying therapeutic proteins throughout the brain of our best rodent models of AD [1]. They generated murine NSC lines that overexpressed neprilysin, and subsequently transplanted these cells into transgenic models [1]. The NSCs survived and continued to function for at least several months post transplantation, they markedly reduced the Aβ pathology associated with AD, and they enhanced synaptic connectivity [1]. This exciting work brings us one step closer to advancing NSC-based biologics into therapies for AD. However, it is important to note that the commonly used human whole fetal graft [7], or NSCs derived from fetal tissue, would not be immunologically matched to the recipient, as it was in the study by Blurton-Jones and colleagues. It is unclear how effective this allogeneic transplantation would be in a somewhat immuno-privileged area, such as the brain. However, recent research demonstrates that significantly higher numbers of autologous (as opposed to allogeneic) neurons have survived in the primate brain following transplantation [8]. A potential solution to this immune rejection problem would be the lifelong administration of immunosuppressive drugs to the patient in order to minimize the immune response in the brain. However, immunosuppressive drugs are expensive, inconvenient, and toxic. Moreover, previous research suggests that they do not appear to significantly improve allogeneic graft survival and may even reduce it [7], making the proposed immunosuppressive drug solution less than ideal.

An alternative approach, which has become increasingly feasible in light of recent discoveries, would be to generate autologous NSCs from human induced pluripotent stem cells (hiPSCs), which themselves have been derived from suitable patient cells, such as skin cells (Figure 1). These cells could be genetically modified in a manner similar to the NSC modification process implemented by Blurton-Jones and colleagues. However, there are concerns that randomly integrated viral vectors (as used in approaches such as the study by Blurton-Jones and colleagues) may induce insertional mutagenesis in a subset of cells, which then would present an increased neoplasm risk [9]. The optimal method for genetic modification of hiPSC-derived NSCs would therefore probably involve avoiding this randomly integrated viral vector approach.

**Figure 1:**
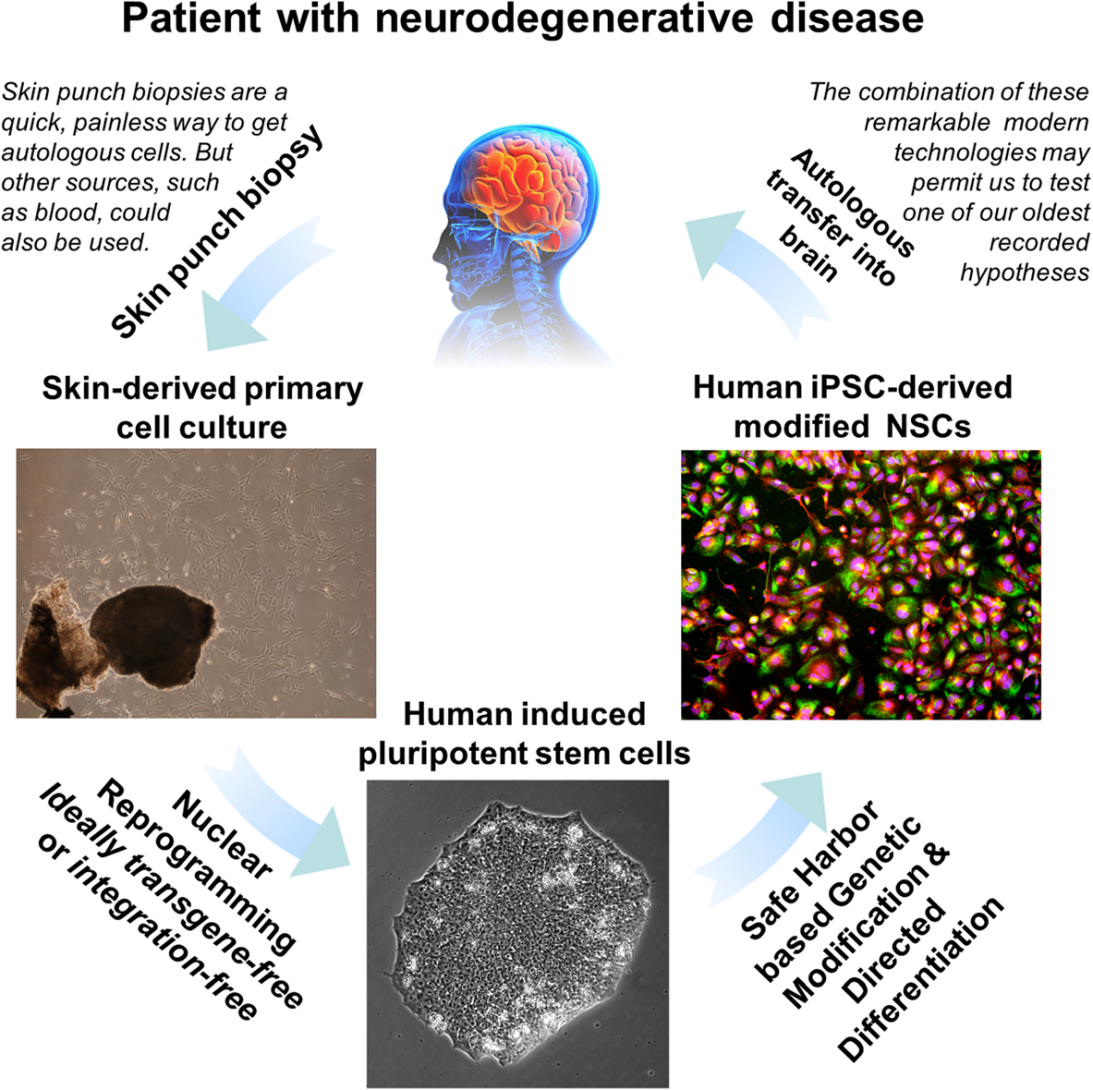
**Developing the neural stem cell-based approach for treating Alzheimer’s disease.** The concept of using human induced pluripotent stem cells (iPSCs) and their neural stem cell (NSC) derivatives, alongside safe-harbor targeted genetic engineering, to develop a personalized cellular therapy for Alzheimer’s disease. Clinically relevant human iPSCs (passage 7) were derived from skin punch biopsies, differentiated into NSCs, and (as shown on the right) stained Pax6 (green), Sox1 (red) and DAPI (blue). The figure is based on unpublished data of Kaitlin Ingraham, Patricia Phelps, and James Byrne. See JA Byrne, unpublished data, 2014 and [10].

One solution to this insertional mutagenesis problem would be the use of powerful, flexible, and inexpensive genome-editing technologies, such as the recently discovered CRISPR/Cas9 system, for safe-harbor-based targeting in hiPSCs. It is anticipated that this safe-harbor framework will soon enable scientists and clinicians to generate hiPSC-NSCs that are genetically modified to express almost any other therapeutically relevant factor without risking random insertional mutagenesis. Recent reports indicate that implanted iPSC-derived NSCs have demonstrated the ability to survive, migrate, and differentiate and to restore lost neurological function [11]. Such reports and the astonishing pace of stem cell-based regenerative medicine provide hope that new advances will continue to resolve the clinical hurdles faced by hiPSC-based therapeutics for neurodegenerative diseases [9].

In conclusion, modified NSCs may represent a critically needed solution to the inevitably increasing prevalence of AD in contemporary society.

## Abbreviations

AD: Alzheimer’s disease; Aβ: Amyloid-beta; hiPSC: Human induced pluripotent stem cell; NSC: Neural stem cell.

## Competing interests

The author declares that he has no competing interests.
